# Bliss and Loewe interaction analyses of clinically relevant drug combinations in human colon cancer cell lines reveal complex patterns of synergy and antagonism

**DOI:** 10.18632/oncotarget.21895

**Published:** 2017-10-19

**Authors:** Muhammad Kashif, Claes Andersson, Sharmineh Mansoori, Rolf Larsson, Peter Nygren, Mats G. Gustafsson

**Affiliations:** ^1^ Department of Medical Sciences, Cancer Pharmacology, and Computational Medicine, Uppsala University Academic Hospital, Uppsala, Sweden; ^2^ Department of Immunology, Genetics, and Pathology, Uppsala University, Uppsala, Sweden; ^3^ Current/Present address: Department of Biosciences and Nutrition, Karolinska Institute, Stockholm, Sweden

**Keywords:** synergy analysis, combinations, iso-genic, COMBIA, R package

## Abstract

We analyzed survival effects for 15 different pairs of clinically relevant anti-cancer drugs in three iso-genic pairs of human colorectal cancer carcinoma cell lines, by applying for the first time our novel software (R package) called COMBIA. In our experiments iso-genic pairs of cell lines were used, differing only with respect to a single clinically important *KRAS* or *BRAF* mutation. Frequently, concentration dependent but mutation independent joint Bliss and Loewe synergy/antagonism was found statistically significant. Four combinations were found synergistic/antagonistic specifically to the parental (harboring *KRAS* or *BRAF* mutation) cell line of the corresponding iso-genic cell lines pair.

COMBIA offers considerable improvements over established software for synergy analysis such as MacSynergy™ II as it includes both Bliss (independence) and Loewe (additivity) analyses, together with a tailored non-parametric statistical analysis employing heteroscedasticity, controlled resampling, and global (omnibus) testing.

In many cases Loewe analyses found significant synergistic as well as antagonistic effects in a cell line at different concentrations of a tested drug combination. By contrast, Bliss analysis found only one type of significant effect per cell line.

In conclusion, the integrated Bliss and Loewe interaction analysis based on non-parametric statistics may provide more robust interaction analyses and reveal complex patterns of synergy and antagonism.

## INTRODUCTION

New targeted cancer drugs designed to interfere with specific signal transduction pathways [1] provide oncologists with new tools for cancer treatment but progress in anti-cancer pharmacotherapy is still very limited in terms of long time survival [2, 3]. Most cancer drug treatments in use are combination regiments developed by adding compounds with novel mechanisms of action to drug treatments in use.

This current selection of anti-cancer drug combinations for use in the clinic is mostly based on conceptually simple theoretical models of the underlying biochemical processes involved assuming synergistic or at least additive anti-tumour effects will emerge when combining established cancer drugs [2]. As illustrated by the recent negative phase III trial investigating the addition of sunitinib to 5-FU and irinotecan in 1^st^ line treatment of advanced colorectal cancer carcinoma (CRC) [4], this empirical ad hoc selection of suitable drug combinations is costly and error prone. Therefore it should preferably be substituted with a strategy based on knowledge gained from more comprehensive preclinical investigations. Thus, there is a great need for more comprehensive and sophisticated preclinical drug combination analyses and CRC *in vitro* models that more successfully can guide the selection of drug combinations suitable for a clinical testing [2, 5].

CRC is a major solid tumour cancer type globally and until a few decades ago there was only one main drug available for treatment, the antimetabolite and thymidylate-synthase inhibitor 5-FU. Since then topoisomerase inhibitor irinotecan and the platinum oxaliplatin have been added to the arsenal of cytotoxic drugs together with the 5-FU analogue capecitabine. These drugs are now often combined to doublet or triplet regimens in the treatment of advanced disease. Such combinations provide better anti-tumour effects than single drugs, mostly in terms of tumour response rates and progression free and overall survival [6].

When it comes to targeted drugs directed specifically towards the tumour cells in CRC, the EGFR antibodies cetuximab and panitumumab are now often routinely added to the chemotherapy in advanced CRC, if the tumour cells are not harboring any *KRAS* or *BRAF* mutation. The tyrosine kinase inhibitor (TKI) regorafenib was recently found to slightly prolong life in advanced CRC refractory to standard drugs [7] and other TKIs targeting the EGFR signal pathway (erlotinib) or multiple signal transduction pathways (sunitinib and sorafenib) are available and have been evaluated in clinical trials as single drugs or combined with the established cytotoxic drugs in advanced CRC.

Based on the current situation we used preclinical *in vitro* cell line models of human CRC to study combinations of standard cytotoxic drugs used for CRC treatment or combined with the small molecule TKI inhibitors erlotinib, sorafenib and sunitinib as well as the experimental drug VLX600 which is now in phase 1 clinical trial. VLX600 causes mitochondrial dysfunction of the metabolically stressed tumor cells leading to bioenergetics catastrophe and cell death [28]. The cell line models used were selected to reflect clinically relevant prognostic and/or predictive molecular status in this cancer type and consisted of three iso-genic pairs, each pair consisting of one parental cell line having a *KRAS* or *BRAF* mutation and one cell line with this mutation knocked out. For example in the iso-genic cell line pair HCT116 + HCT116KRAS/-, HCT116 has mutated *KRAS* gene and this cell line is parental to HCT116KRAS/- that has *KRAS* mutated allele knocked out. The synergy analyses performed on the experimental data by using a novel software (R package) developed in-house called COMBIA (COMBination Interaction Analysis). It provides both Bliss and Loewe analyses and does not require any manual data entry making it fit for automated drug discovery pipelines. COMBIA can be installed directly from CRAN (The Comprehensive R Archive Network, https://cran.r-project.org/).

COMBIA was developed to offer several improvements relative to established commercial tools like MacSynergy™ II [8], which has been cited in 150 peer reviewed articles [9], and the even more widely used and cited software CompuSyn (earlier known as CalcuSyn). Notably the two versions of MacSynergy™ II available only offer either Bliss (independence) [10] or Loewe [11, 12] (additivity) synergy/antagonism analysis. Similarly, CompuSyn only offers a family of combination indices [13] rooted in enzyme kinetics for the same task but most users only use the combination index which corresponds to conventional Loewe analysis. By contrast COMBIA provides both Bliss and Loewe analyses and it does not need any manual data entry.

There are already a few other R packages available for combination synergy/antagonism calculations. The package mixlow implements the Loewe additivity model [11, 12] for synergy/antagonism calculation [14] and the package hbim uses the Bliss independence model [10] for calculation of synergy while focusing on vaccines [15]. R package synergyfinder is offering analyses according to multiple models [30]. As further elaborated below, COMBIA seems to be the first to offer all the following five features in a single package/software: (1) Freely available as open source. (2) Enables synergy analyses according to both Bliss (independence) and Loewe (additivity). (3) Offers robustness against outliers. (4) Takes heteroscedasticity (survival level dependent experimental variability) into account by selecting a relevant subset of residuals for resampling statistics. (5) Performs non-parametric resampling (bootstrapping) based synergy analysis that makes general/weak a priori assumptions regarding the statistical distribution of the experimental variability.

As shown and discussed below together with additional findings, the joint Bliss and Loewe synergy analyses performed using COMBIA suggests that 2 of the 15 combinations tested may offer mutation specific synergy and further 2 combinations may offer mutation specific antagonism. However, instances of mutation non-specific synergy/antagonism were also found. Concentration dependent synergies, both specific and non-specific, offered by these combinations suggest their further development by maintaining synergistic concentration ratios *in vivo* perhaps by using already reported technologies [16, 17].

## RESULTS

Analyses were performed to identify synergism/antagonism suggested by joint Bliss and Loewe analyses as presented in first subsection for all the drug pairs and cell lines studied, followed by a summary (Table 1) pinpointing the interactions found to be non-specific second subsection or specific third subsection to *KRAS/BRAF* mutations. Thus in second subsection results regarding drug pairs showing synergy and/or antagonism regardless of *KRAS/BRAF* mutation status are presented. Then in third subsection, interactions that are found specific to the parental cell line (harboring *KRAS/BRAF* mutation) in the corresponding iso-genic cell line pair are extracted. In context of this article, a combination is said to be non-specific if it is synergistic/antagonistic in at-least two cell lines without any respect to mutation status of these cell lines. For example, in Table 1, combination erlotinib + irinotecan is non-specific because it is synergistic to the three cell lines HCT116, HCT116KRAS/- and DLD-1KRAS/-. Two of these cell lines have a mutated *KRAS* gene and one with this mutation knocked out. Analogously, a combination is said to be specific if it exhibit synergy/antagonism only in the parental cell line of an iso-genic pair. As an example, also according to Table 1, combination VLX600 + oxaliplatin is synergistic specifically to the *KRAS* mutated cell line HCT116 in the iso-genic cell line pair HCT116 + HCT116KRAS/-.

**Table 1:** Summary of joint Bliss and Loewe synergy and antagonism analyses of drug pairs when exposed to 6 different colorectal cell lines, one for each column

Combination	HCT116	HCT116KRAS/-	DLD-1	DLD-1KRAS/-	RKO	RKOBRAF/-/-
Erlotinib & Sorafenib	--	--	--	--	--	--
Erlotinib & Sunitinib	**O**	**O**	**X**	**X**	--	--
5FU & Irinotecan	--	--	--	--	--	--
5FU & Oxaliplatin	--	**O**	**O**	--	**O**	--
Sorafenib & 5FU	--	--	--	--	--	--
Sorafenib & Irinotecan	--	--	--	--	--	--
Sorafenib & Oxaliplatin	--	--	--	--	--	--
Sunitinib & Irinotecan	--	--	--	--	--	--
Sunitinib & Oxaliplatin	--	--	--	**O**	--	**O**
Sunitinib & 5FU	**O**	--	--	--	NA	**O**
Erlotinib & 5FU	--	**X**	--	--	--	--
Erlotinib & Irinotecan	**X**	**X**	NA	**X**	--	--
Erlotinib & Oxaliplatin	--	--	**O**	NA	**O**	**O**
VLX600 & Irinotecan	--	--	**X**	--	NA	--
VLX600 & Oxaliplatin	**X**	--	--	--	--	--

Each element in the table, which corresponds to one particular drug combination and cell line, is marked by “X” in case there exists a joint Bliss and Loewe synergy (occurs when the bootstrap interval of *I*_*max*_ under the Bliss or Loewe null model does not include observed *I*_*max*_), and by “O” if there exists a joint Bliss and Loewe antagonism (occurs when bootstrap interval of *I*_*min*_ does not include observed *I*_*min*_). Symbol “--” represents combinations where no global joint Bliss and Loewe synergy or antagonism exists. For the cases where there is no data collected or where Loewe analysis cannot be performed (due to an almost constant concentration-response relationship according to the experimental data collected) the element is marked “NA”. Detailed summary results for each Bliss and Loewe analysis are shown in Supplementary Table 1 and Supplementary Table 2.

A summary of synergy and antagonism across all experiments is shown in Table 1, detailed summary results for each Bliss and Loewe analyses are shown in Supplementary Table 1 and Supplementary Table 2. Each element in Table 1, which corresponds to one particular drug combination tested on a cell line, is marked by “**X**” in case there exists joint Bliss and Loewe synergy (occurs when the BIs of *I*_*max*_ under the Bliss and Loewe null models do not include observed *I*_*max*_), and by “**O**” for joint Bliss and Loewe antagonism (occurs when the BIs of *I*_*min*_ do not include observed *I*_*min*_). The symbol “--” represents a combination where no global joint Bliss and Loewe synergy, or antagonism, was detected. For the cases when no data were collected or Loewe analysis could not be performed (due to an almost constant concentration-response relationship according to the experimental data collected), the element is marked by “NA”.

### Joint Bliss and Loewe synergy/antagonism analyses

**Figure 1:**
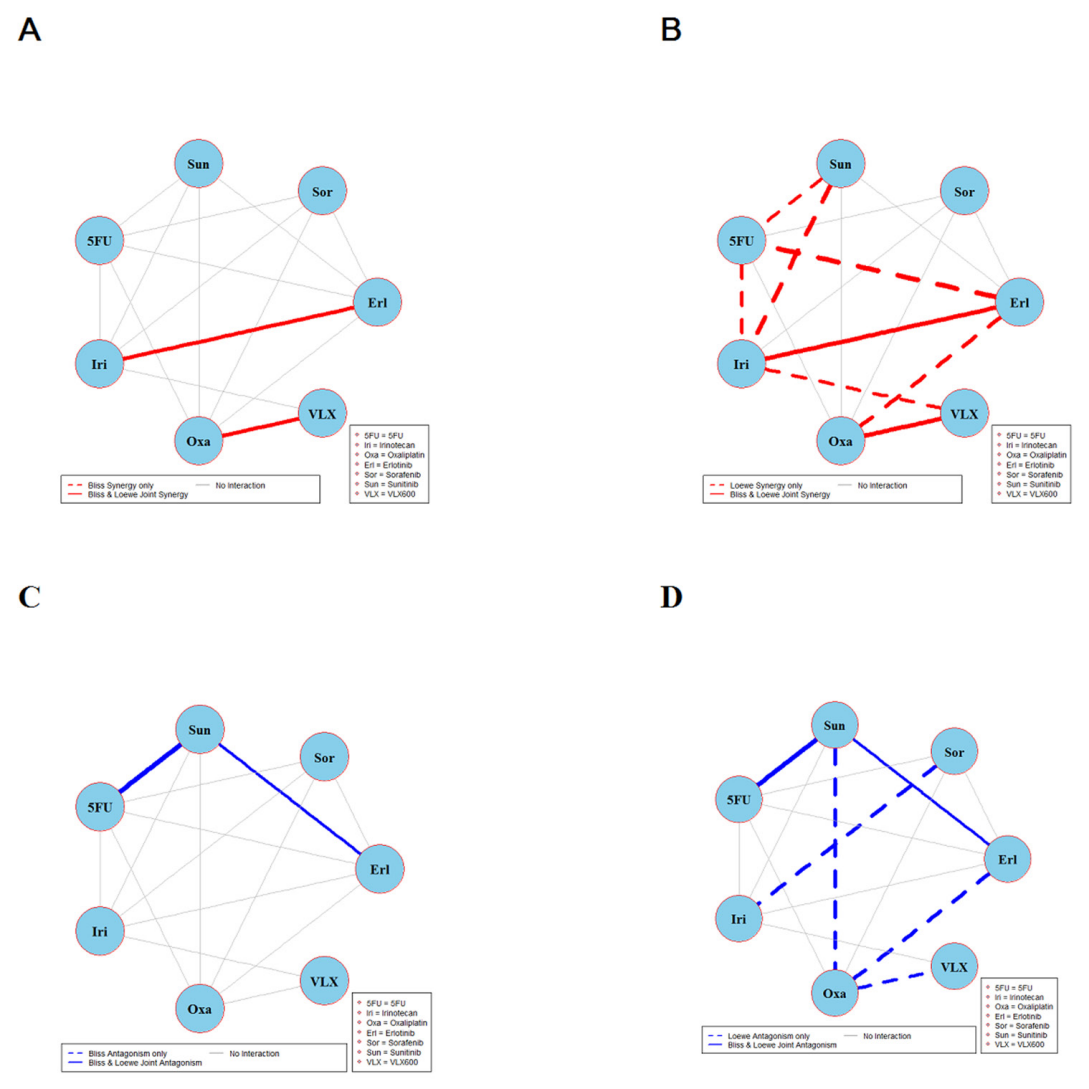
Global synergy/antagonism analyses by using both Bliss and Loewe models in HCT116 cell line Panels **(A/B)** presents synergy analysis according to Bliss and Loewe models and panels **(C/D)** presents the antagonistic analysis as per the Bliss and Loewe models. In panel B dotted line indicates synergy according to the Loewe model only whereas a dotted blue line in panel D indicates antagonism according only to Loewe. Solid lines represent the synergy or antagonism as per joint Loewe and Bliss analysis. The width of each colored line represents the total sum of synergy indices calculated for individual (well specific) concentration combinations. Notably the width may be different for Bliss (left) and Loewe (right). A thin gray color line indicates that the corresponding drug pair showed no global synergy or antagonism.

**Figure 2:**
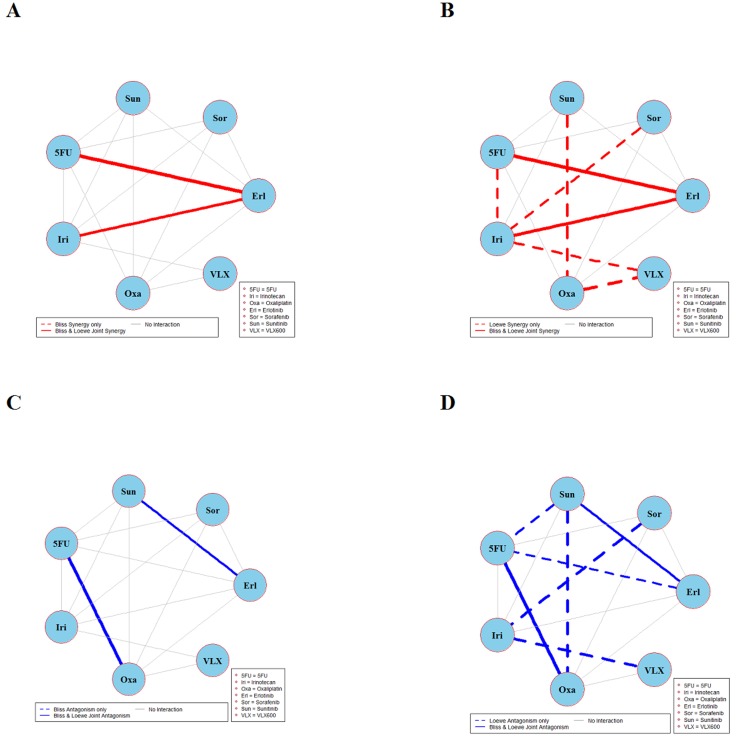
Global synergy/antagonism analyses by using both Bliss and Loewe models in HCT116KRAS/- cell line Panels **(A/B)** presents synergy analysis according to Bliss and Loewe models and panels **(C/D)** presents the antagonistic analysis as per the Bliss and Loewe models.

**Figure 3:**
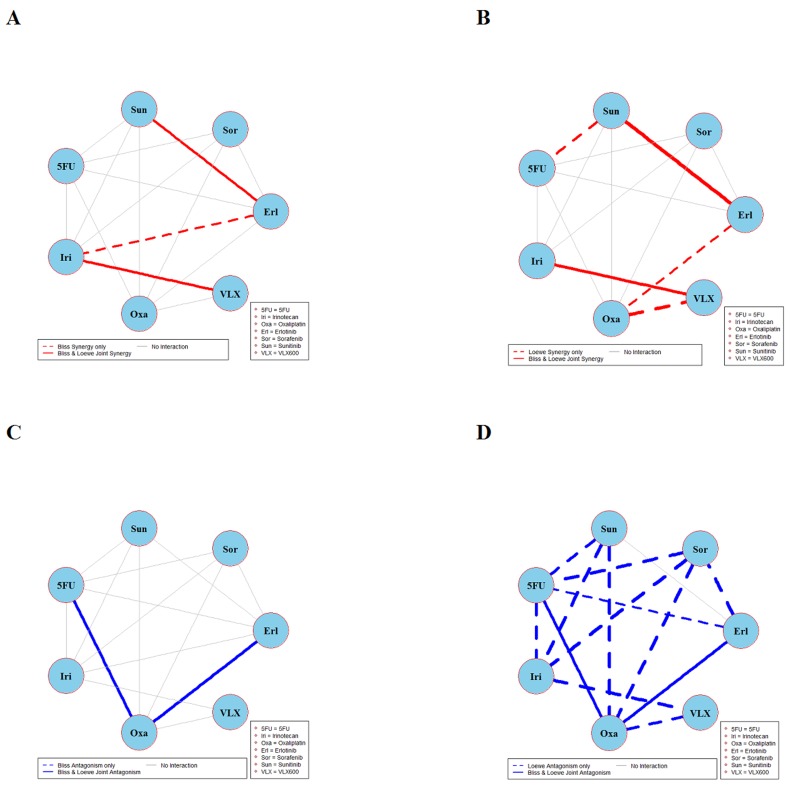
Global synergy/antagonism analyses by using both Bliss and Loewe models in the DLD-1 cell line Panels **(A)** and **(B)** present results from synergy analysis where a solid red line indicates synergy according to both Bliss and Loewe and dotted lines indicate synergy according only to Bliss or Loewe model. Panels **(C)** and **(D)** presents corresponding results from an analysis of antagonism.

**Figure 4:**
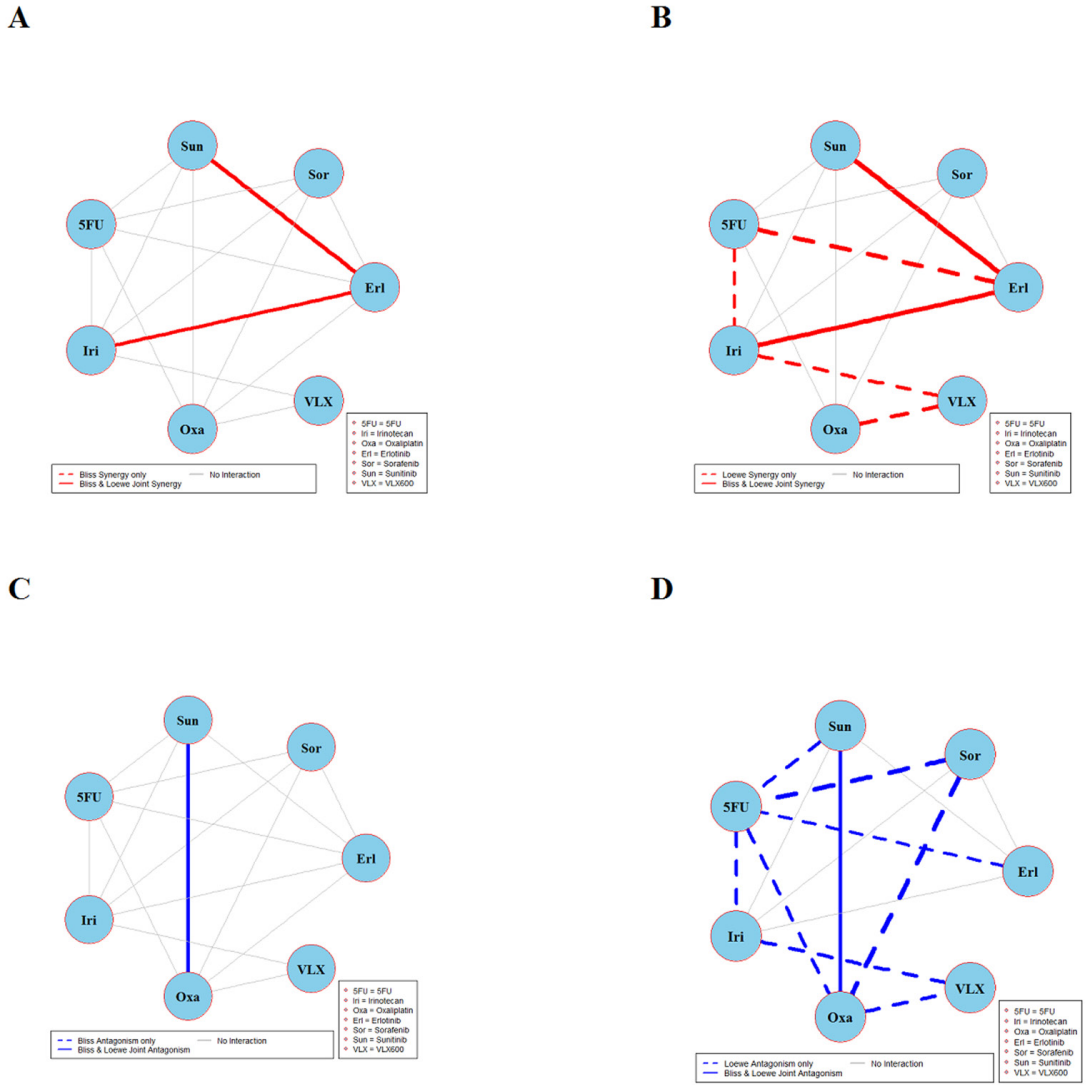
Global synergy/antagonism analyses by using both Bliss and Loewe models in DLD-1KRAS/- cell line Panels **(A/B)** presents synergy analysis according to Bliss and Loewe models and panels **(C/D)** presents the antagonistic analysis as per the Bliss and Loewe models.

**Figure 5:**
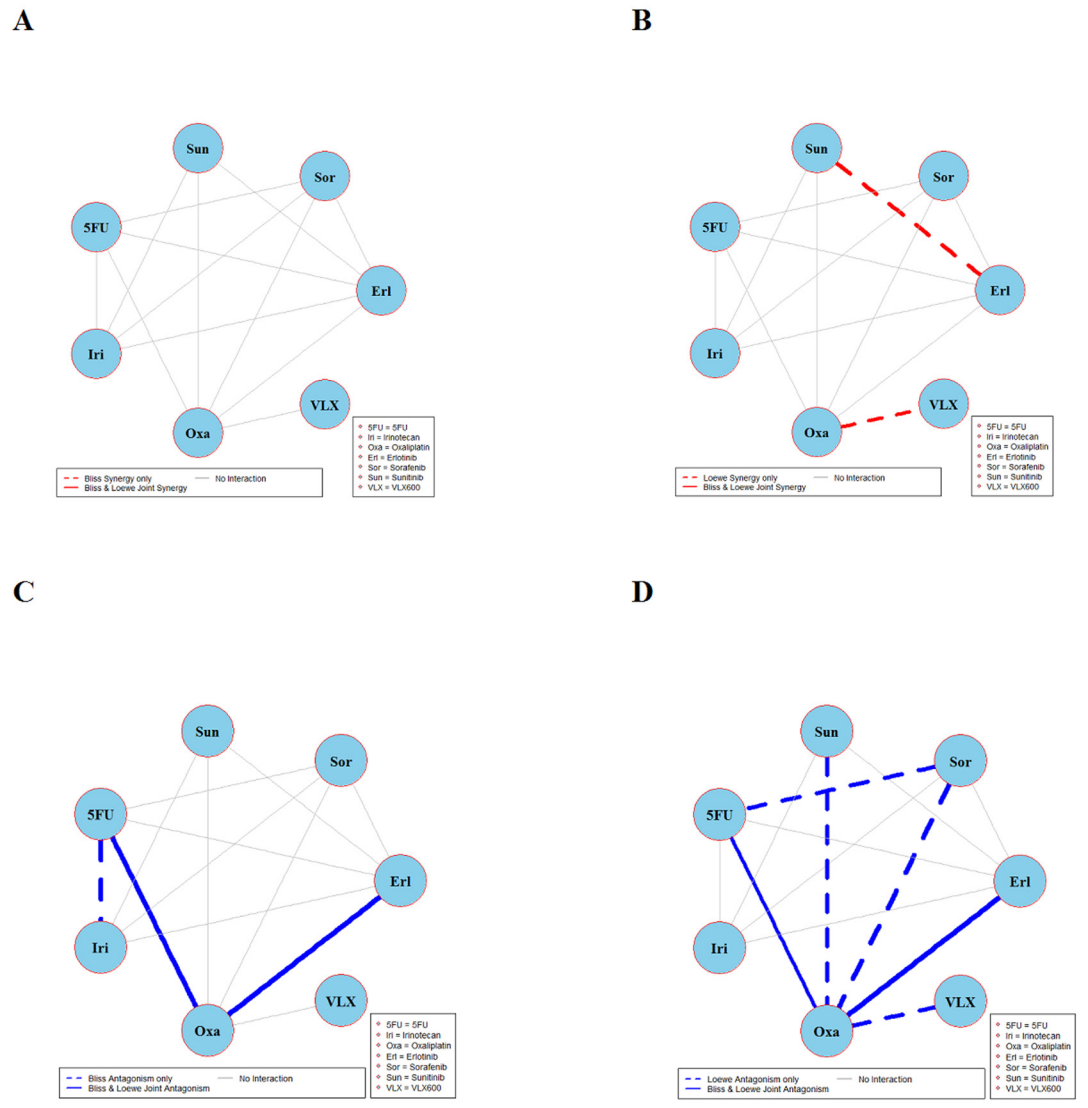
Global synergy/antagonism analyses by using both Bliss and Loewe models in RKO cell line Panels **(A/B)** present synergy analysis according to Bliss and Loewe models. It can be seen in panel A that Bliss model did not find any significant synergistic combination. However, Loewe analysis in panel B found two synergistic combinations represented by dotted red lines. Panels **(C/D)** presents the antagonistic analysis as per the Bliss and Loewe models.

**Figure 6:**
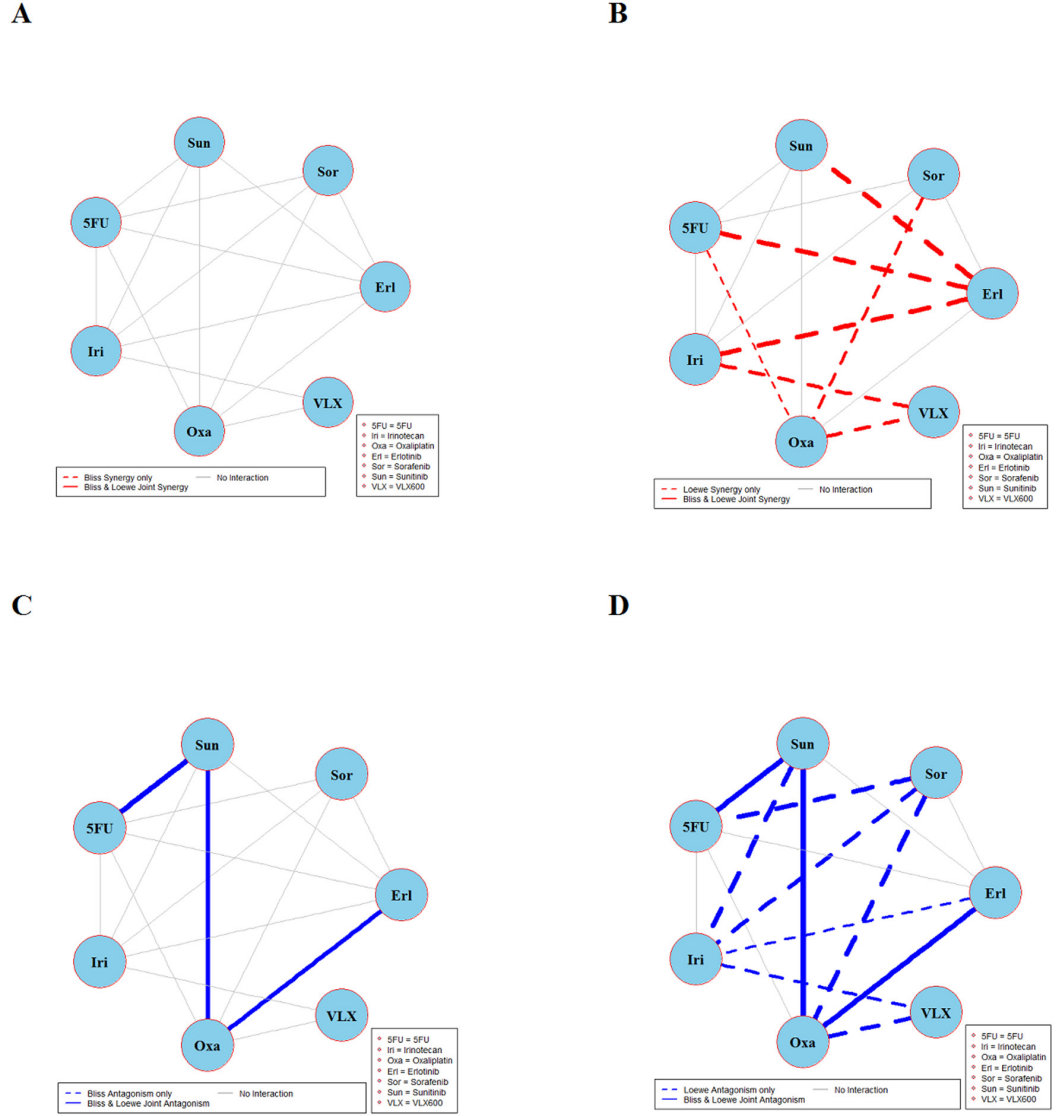
Global synergy/antagonism analyses by using both Bliss and Loewe models in RKOBRAF/-/- cell line Panels **(A)** and **(B)** present synergy analyses according to Bliss and Loewe models and panels **(C/D)** presents the corresponding antagonistic analyses as per Bliss and Loewe models.

The results of global testing for synergistic and antagonistic interactions in all six cell lines are presented in Figures 1-6. In each panel, drugs are represented as circles and each tested drug pair is represented by a line that connects the corresponding circles. A red solid line indicates detection of synergy (defined as, observed *I*_*max*_ is larger than the upper bound of bootstrap interval for *I*_*max*_), according to both Loewe and Bliss models, using the global (omnibus) test. A red dotted line indicates that synergy is present according to only one of the two synergy models: according to the Bliss model shown only in panel A and according to the Loewe model only in panel B. Similarly, solid lines in panels C & D colored blue indicate antagonism (defined as, observed *I*_*min*_ is smaller than lower bound of bootstrap interval for *I*_*min*_), according to both Loewe and Bliss. The blue dotted lines indicate evidence of antagonism either according only to Bliss (panel C) or only to Loewe (panel D). The width of each colored line represents the total sum of synergy indices calculated for individual (well specific) concentration combinations. Notably the width may be different for Bliss (left) and Loewe (right). A thin gray color line indicates that the corresponding drug pair shows neither global synergy nor antagonism.

Figure 1 shows the combination interactions in the CRC cell line HCT116. Panel A shows that both Bliss and Loewe synergies are present in the two combinations erlotinib + irinotecan and oxaliplatin + VLX600, represented by the red solid line. In the same panel there are 13 additional combinations that don’t show any Bliss synergy (gray lines). In panel B the corresponding results based on Loewe synergy analyses are presented. It can be seen that according to Bliss, only two combinations are detected as synergistic in panel A while the Loewe analysis in panel B show eight synergistic combinations (red solid and dotted lines). In the same panel there are 7 combinations that are not showing synergy. Analogous results for antagonistic interactions are shown in panels C and D. Here two combinations show various degrees of joint Bliss and Loewe antagonism. All 4 combinations represented by solid lines (joint Bliss and Loewe synergy or antagonism) are decomposed at their respective concentrations and shown in Supplementary Figure 1.

Analyses of combination interactions in HCT116KRAS/- are presented in Figure 2. Two combinations demonstrate joint Bliss and Loewe synergy (see the red solid lines in panels A and B). In panels C and D two combinations show antagonism. See Supplementary Figure 2 for details about the concentrations where these combinations are synergistic/antagonistic.

Combination interaction results in the DLD-1 cell line are presented in Figure 3. The two combinations irinotecan + VLX600 and sunitinib + erlotinib are synergistic according to joint Bliss and Loewe synergy analyses and shown in panels A and B. In panels C and D, combinations 5FU + oxaliplatin and erlotinib + oxaliplatin are antagonistic. Notably there are only 14 connected lines (combinations) in panels B and D as compared to 15 in panels A and C. This is because Loewe synergy analysis cannot be performed for one of the 15 combinations performed because the corresponding data collected cannot provide reliable estimates of the two concentration-response curves required. See Supplementary Figure 3 for detailed results of combinations irinotecan + VLX600, sunitinib + erlotinib, 5FU + oxaliplatin, and erlotinib + oxaliplatin.

Figure 4 depicts the synergy/antagonism analyses of 15 combinations in the DLD-1KRAS/- cell line. The joint Bliss and Loewe synergy analyses identified two synergistic (panels A and B) and one antagonistic combination (panels C and D), see Supplementary Figure 4 for details. The synergy/antagonism analyses for the RKO cell line are shown in Figure 5. Joint Bliss and Loewe synergy analyses identified no synergistic but two antagonistic combinations, see panels A, B and C, D. For details see Supplementary Figure 5. RKOBRAF/-/- cell line synergy/antagonism analyses are shown in Figure 6. No combination was identified synergistic according to joint Bliss and Loewe synergy analyses shown in panels A and B. 3 antagonistic combinations were identified according to joint Bliss and Loewe antagonism analyses these are shown in panels C and D. Details of these combinations are presented in the Supplementary Figure 6.

### Non-specific joint synergistic/antagonistic interactions

From Table 1, it can be seen that the combination erlotinib + sunitinib is synergistic, non-specifically, in DLD-1 and DLD-1KRAS/- cell lines but this combination is antagonistic in the HCT116 and HCT116KRAS/- cell lines. From Table 1, it can also be found that combination erlotinib + irinotecan is synergistic in three cell lines. One also finds that four combinations (5FU + oxaliplatin, sunitinib + oxaliplatin, sunitinib + 5FU and erlotinib + oxaliplatin) are antagonistic non-specifically in two or more cell lines and therefore less promising for clinical use.

### Specific joint synergistic/antagonistic interactions

In Table 1, it is shown that combinations VLX600 + irinotecan and VLX600 + oxaliplatin are synergistic especially to *KRAS* mutated cell lines in DLD-1 + DLD-1KRAS/- and HCT116 + HCT116KRAS/- cell line pairs, respectively. Combinations 5FU + oxaliplatin is specifically antagonistic in DLD-1 + DLDKRAS/- and RKO + RKOBRAF/-/- cell line pairs, while combination sunitinib + 5FU shows specific antagonism in HCT116 + HCT116KRAS/- pair.

## DISCUSSION

### COMBIA: A novel freely available R-package

The open source R package COMBIA used for the first time in this work is novel and available at comprehensive R archive network (http://cran.r-project.org/). This package performs synergy/antagonism analyses of drug combinations based on the Bliss independence and the Loewe additivity models. The package is able to save analyzed data and graphs on the user machine, ready for use in scientific publications. COMBIA does not require manual data entry; data can be directly input from different plate readers in user defined formats. For more details see the user documentation of COMBIA.

In addition to limitations regarding the different types of synergy/antagonism analyses offered by the different packages/software already available, in most cases the statistical analyses provided are quite limited as they are based on assuming normal distributions for the experimental variability as well as constant experimental variability regardless of effect level. As a consequence, the analyses are not accurate when experimental variability cannot be approximated by a normal distribution and when the experimental variability depends on the effect level. How often this occurs is of course application dependent and generally unknown. Although there are well established statistical tests to determine if a set of observations seems to be drawn from a normal distribution or not, this is not yet implemented in those softwares. Anyhow, such a solution would of course not be useful in all the cases when the statistical test used suggests that the variability deviates from being normally distributed.

Moreover, these packages/softwares do not come with any safeguard against outliers, leaving it up to the user to take care of them before synergy/antagonism analyses. In COMBIA these issues have been addressed by means of automatic analysis/rejection of outliers and by a non-parametric statistical analysis based on resampling (bootstrapping) making only weak/general assumptions of the statistical distribution of the experimental variability. Notably, COMBIA also takes care of heteroscedasticity in experimental variability by employing an adaptive local pool of relevant residual errors reflecting the current effect level. All these features render COMBIA fit stand alone as well as part of high-throughput combination drug discovery pipelines.

During the work reported here, we noted that the statistical analysis offered in MacSynergy™ II, which uses normal distribution statistics, is in fact neither described nor implemented properly. In particular, the threshold value used to determine if the difference between observed and predicted effects for a particular concentration combination is greater than zero is simply set to 1.96d where d denotes the standard deviation for the collected data for the actual drug combination. Thus the standard deviations related to the single drug responses, which are used to calculate the expected combination response, are ignored. In the MacSynergy™ II manual (p.39) it is stated that “This approach is unacceptable to some statisticians”. Moreover, there is no adjustment for multiple testing included. Bonferroni correction is mentioned in the manual but in a different context and never used in the synergy calculations. In particular, it is stated in the manual that (p 39) that “MacSynergy™ II gives the statistics for significant synergy at 95%, 99% and 99.9% confidence. The other statistical parameter provided is the confidence level calculated with a Bonferroni adjustment. This is claimed in the manual to calculate the probability that “we have correctly and simultaneously identified every value in the matrix as statistically significant”. Clearly the potential role of Bonferroni adjustment is not correctly explained as the idea of compensating for multiple testing is not properly described. Finally, it is not obvious that the 95% confidence interval provided by MacSynergy™ II for a global synergy value (sum of observed positive differences across all concentration combinations for a given drug pair) has any solid theoretical foundation, in particular since it does not come with any explicit use of standard deviations but implicitly relies on the already mentioned threshold value used for detection of significance. These mistakes are serious as they make any statistical synergy analysis provided by MacSynergy™ II invalid, even when the experimental variability is normally distributed. Thus the many previously reported synergy results obtained using MacSynergy™ II should be reconsidered to avoid misleading conclusions.

### Non-specific synergy

It would be of great clinical interest to identify drug pairs that are synergistic regardless of the *KRAS/BRAF* mutations. These combinations would be more generally applicable than combinations only active specific to the *KRAS/BRAF* mutations. Among the 15 drug pairs studied here, two of them showed such non-specific synergy in at least two cell lines and there are five combinations that are antagonistic in two or more cell lines (for details see Figures 1-6, Table 1, and supplementary material). Further studies are required, especially for the synergistic combination of erlotinib + irinotecan, to determine the generality of these results in other *in vitro* model systems including early passages of extracted patient tumour cells.

### Specific synergy

Combination treatment of CRC and other solid tumours is an important strategy to optimize the use of the available drugs. In the work reported here a study of 15 clinically relevant drug combinations in three different iso-genic cell lines harboring clinically relevant differences in *KRAS* and *BRAF* status was performed. According to joint Loewe and Bliss analyses two combinations, VLX600 + irinotecan and VLX600 + oxaliplatin, were found to be synergistic to *KRAS* mutated cell lines in the two iso-genic cell line pairs DLD-1 + DLD-1KRAS and HCT116 + HCT116KRAS. However, further studies are required for characterization of these results.

### Bliss and Loewe do not always agree

As explained by others and in the supplement of our previous work [18], synergy is said to occur when the effect of a dose mixture is larger than ‘expected’ according to the particular (Loewe or Bliss) model employed. Therefore, as found here, in practice it is possible to end up with seemingly confusing situations. For example, a trivial case where one drug is mixed with a diluted version of itself may result in detection of synergy according to Bliss while this is the canonical example of Loewe additivity. Similarly, a trivial multiplicative effect that would be ignored by a Bliss analysis could be detected as antagonistic in a corresponding Loewe analysis. There is also one special case for which neither a Loewe nor a Bliss analysis would suggest synergy as illustrated by the following example^18^. Assume two very different drugs A and B that are affecting the cell survival independently via two unrelated and very different cell surface receptors, one for each drug. Also assume that locally, both drugs have exponential dose-response relationships,$R\left(c_{aA}\right)=e^{\alpha c_{A}}$ and $R\left(c_{B}\right)=e^{\beta c_{B}}$ , in some concentration ranges of interest. Then the resulting Bliss prediction is $R_{Bliss}\left(c_{A},c_{B}\right)=e^{\alpha c_{A}+\beta c_{B}}$ which corresponds to linear isoboles (Loewe additivity). Thus, while the drugs have independent contributions in this special case they also *behave* as being diluted versions of each other. Similarly, if two drugs with exponential concentration-response relationships would act as diluted version of each other, they would also *behave* as acting independently of each other.

In conclusion, conventional detection of synergy is always related to unexpected deviations from a particular pre-defined effect model that is considered biologically/mechanistically trivial and therefore uninteresting like drugs showing independent actions (Bliss) and/or acting as diluted versions of each other (Loewe). As deviations from independence and additivity are not expected to occur simultaneously, one simple approach is to collect all drug combinations that show synergy regardless of the pre-defined effect model used. A perhaps more natural and stringent approach is to keep only those drug combinations that deviate from all types of trivial pre-defined effect models. Employing this practice in the study presented here we report two combinations VLX600 + irinotecan and VLX600 + oxaliplatin, which may be showing *KRAS/BRAF* mutation specific synergy according to both Bliss and Loewe models. Another drug combination, erlotinib + irinotecan, induces non-specific synergy, irrespectively of a *KRAS/BRAF* mutation status in three cell lines. It seems suitable that these combinations may be explored further *in vivo*.

### Local concentration regions of synergistic and antagonistic interactions

As already reported previously by others [16, 17, 19], our results also show that synergy does not occur across a wide range of concentration combinations for the drug pairs and *in vitro* models studied. Indeed the same drug combination can produce synergy at one range of concentration combinations while also being antagonistic at another range of concentration combinations. Sequence of drug treatment in a combination and drugs exposure times are still other factors adding complexity to clinical situations. However, as already reported [16, 17], these complexities might be overcome *in vivo* by maintaining the drug concentrations/ratios for example by using special delivery systems [16]. This also suggests that instead of using drug concentrations corresponding to the maximum tolerated *in vivo* one may select concentrations that are guided by *in vitro* results [16].

### Potential limitations

#### Dual synergy and antagonism patterns

Dual synergy and antagonism patterns are identified in some of the tested combinations. In most cases synergy and antagonism are found at different concentration ranges of a drug combination. As already explained in the previous subsection above, this observation has support from other studies [16, 17, 19] and here this observation is the result of a rigorous omnibus statistical testing procedure applied using the conventional 5% threshold for the bootstrap intervals obtained. As for all statistical tests, it is important to remember that our test results do not say anything about effect levels but they show that one cannot reject the possibility that these synergy and antagonism patterns are non-existing. The synergistic and antagonistic effects are often not very large but based on the statistical analyses performed they are indeed statistically significant. To what extent the corresponding effect levels have practical importance is a relevant topic for future work.

#### BRAF targeting compounds

During the study design phase, we consider the inclusion of BRAF targeting compounds but decided against it due to followings reasons: (i) We do target the BRAF pathway by means of sorafenib which has BRAF as one of its targets and indirectly by erlotinib which is inhibiting other parts of the same pathway. (ii) The more specific BRAF inhibitor vemurafenib was considered for this project. However it was not included, partly because its effect on cell viability starts at relatively high concentration in the cell lines used (IC50 is 20-30 μM). Moreover it was not included because is not used clinically as a standard drug for treatment of colorectal cancer. Anyway, it would be interesting to include vemurafenib, and possibly other approved RAF-MEK-ERK pathway inhibitors, in future work as the use of these drugs in combination with others may represent opportunities to expand the indications of them to colorectal cancer. (iii) Clinically, a *BRAF* mutation in colorectal cancer predicts no effect of EGFR inhibitors and also predicts poor clinical outcome [29]. Moreover, BRAF inhibitors are not used as a standard treatment for colorectal cancer.

#### Prodrugs

Given that irinotecan is a prodrug, in future work it would be interesting to test its active metabolite, SN-38. The effects of irinotecan in the cell cultures could in part be dependent on the capabilities of the cells to convert it to active metabolites.

#### More compact figures

It is possible to create more compact figures by merging the results for synergy and antagonism into one figure using different types of lines and colors. However, this was not used in this work as the resulting figures will be very crowded and therefore difficult to grasp (data not shown).

### Clinical usefulness

One should always keep in mind that from a clinical point of view, drug combinations may be useful even if there is no synergistic effect according to Bliss and/or Loewe [18]. This would be the case if drug components do not overlap in normal tissue toxicity, yet have at least some non-shared cytotoxic effect against the tumour cells. Given the results reported here on the complexity of drug interactions and the difficulties in finding robust synergy across wide concentration regions *in vitro*, it seems like the clinical benefits observed using drug combinations instead of single drugs in cancer treatment are due to non-synergistic effects which would have been considered ‘trivial’ according to Bliss and/or Loewe. This also supports the general idea recently re-introduced to replace the synergy analyses according to Bliss and Loewe (which both are rooted in environmental toxicology) by the search for therapeutic synergy simply defined to occur when the combination offers a larger differential activity between cancer cells and normal cells than when any of the single constituent drugs is used alone [18].

## MATERIALS AND METHODS

In total, approximately 6000 different concentration combinations distributed across 15 different two-drug combinations were studied. Each concentration combination experiment was performed four times.

### Cell lines

Three iso-genic CRC human cell line pairs were used. These were HCT116 + HCT116KRAS/-, DLD-1 + DLD-1KRAS/- and RKO + RKOBRAF/-/- (double knockout). The only difference between the cell lines of each pair is the presence of a mutated *KRAS*/*BRAF* allele in one of them (the parental first cell line in the pairs above) known to be associated with lack of effect of EGFR inhibition and/or poor clinical outcome, while this allele has been knocked out in the second cell line in each pair. All cell lines were obtained from Horizon Discovery and were cultured at 5% CO2 and 37°C in McCoy’s 5a medium supplemented with 10% heat-inactivated fetal calf serum, 2mM glutamine, 100μgml^-1^streptomycin and 100Uml^-1^ penicillin.

### Drugs

A total of 7 drugs were used: three cytotoxic drugs 5-FU, irinotecan, oxaliplatin, three targeted drugs erlotinib, sorafenib, sunitinib and one experimental drug VLX600. Cytotoxic drugs were obtained from Sigma-Aldrich, targeted drugs from LC Laboratories and VLX600 was obtained from Vivolux AB (Sweden). 15 out of the 21 possible two-drug combinations one may design using these 7 drugs were tested (Table 1). These 15 were selected based on their use in clinical practice and based on particular research interests.

### Drug exposure and fluorometric microculture cytotoxicity assay (FMCA)

The cell viability assay FMCA [20] was used to measure the cytotoxic effects of drugs and drug combination on the cell lines studied. The experiments were performed in 384-well microtiter plates seeded with 2 500 cells in 50μl culture medium per well for all cell lines. FMCA relies on the fact that hydrolysis of fluorescein diacetate (FDA) in intact cells plasma membranes after 72 h treatment of compound/combination results into fluorescein. The fluorescein produces fluorescence proportional to cell count and is measured using a plate reader. Every microtiter plate containing growing cells has a set of blank wells (that contain only medium) as well as a set of growth control wells (that contain medium and cells). The survival index (S) is calculated as

$$S=\frac{f_{\mathrm{exposed}}-f_{\mathrm{blank}}}{f_{\mathrm{control}}-f_{\mathrm{blank}}}$$

where *f*_*exposed*_ denotes the fluorescence from the drug exposed cells, *f*_*blank*_is the mean fluorescence from the blank wells and *f*_*control*_ is the average fluorescence signal from the wells without added drugs. In order to accept an assay the ratio between *f*_*control*_ and *f*_*blank*_ must be larger than 5 and the coefficient of variation of the control wells as well as replicate exposures must be less than 30%. The assay is repeated if these criteria are not satisfied.

### Calculations performed in COMBIA

Let *S(i,j,k)* denote the survival index measured in well *(i,j)* in microtiter plate *k*, *k=1,2,…K*. Thus the index *i (i=0,1,2,…,I)* corresponds to a particular concentration of drug A (the first drug in the combination studied) while the index *j (j=0,1,2,…,J)* corresponds to a particular concentration of drug B (the second drug). For *i=0* and *j=0* the corresponding concentration of the drug equals zero, thus for example *S(0,2,3)* should be interpreted as the survival index obtained on plate *k=3* when only drug B was present at a concentration corresponding to the index *j=2*. In our own experiments presented here we used *K=4* plates and there were *I=8* different non-zero concentrations of drug A and *J=6* different non-zero concentrations of drug B. The resulting *K=4* matrices of dimension *7x9* containing the survival indices obtained are then used to calculate a matrix of synergy index values *I(i,j)* defined in eq. (3).

#### Removal of outliers

For each batch of *K=4* plates/experiments, outliers were identified and removed as follows. For each concentration combination *(i,j)*, the corresponding coefficient of variation *CV*_*ij*_ was determined. If *CV*_*ij*_*>30%*, then the most extreme observation/observations were removed by removing replicate/replicates with maximal contribution in *CV*_*ij*_. This procedure was repeated as long as *CV*_*ij*_*>30%* and *K > 2*.

#### Bootstrap interval (BI) calculations

Bootstrap intervals were calculated as described below. Both our Loewe and Bliss analyses rely on the empirical distribution of residuals.

#### Residuals

For each well *(i,j)*, *K* different residuals are determined as

(1) $$e\left(i,j,k\right)=S\left(i,j,k\right)-{<S\left(i,j,k\right)>}_{k}\;k=1,2,...,k$$

where *<S(i,j,k)>*_*k*_ simply denotes the average of *S(i,j,k)* across all values of *k*:

(2) $$<S\left(i,j,k\right){>}_{k}=\frac{1}{K}\sum_{k=1}^{k}S\left(i,j,k\right)$$

Notably the empirical distribution of the residuals *e(i,j,k*) may be interpreted as an estimate of the distribution of experimental error.

#### Bliss Bootstrap interval (BI) calculations

##### BIs for each well (i,j) using sliding window of relevant residuals

Because experimental errors are heteroscedastic (i.e. depending on effect level), a local window of 10% immediate neighbors in terms of SI value was created for each well *(i,j)* on plate *k*. A set of residuals *e*_*s*_*(i,j,k)* consisting of 10% of total residuals was calculated such that all residuals *e*_*s*_*(i,j,k)* are drawn exclusively among the 10% immediate neighbors of *<S(i,j,k)>*_*k*_. For example, for well *(0,1)* on plate 1 with *<S(0,1,1)>*_*k*_ =15 and the total number of concentration combinations being equal to 80 (63 true concentration combinations plus 17 collapsed “combinations” with one or both drugs having zero concentration); residuals of 8 (10%) immediate neighbors in terms of having the closest mean value to *<S(0,1,1)>*_*k*_ are used in the process of drawing the value *e*_*s*_*(0,1,1)*.

A Bliss associated synergy index *I*_*Bliss*_*(i,j)* corresponding to well *(i,j)* is defined as

(3) $$I_{Bliss}\left(i,j\right)=<S\left(i,0,k\right){>}_{k}*<S\left(0,j,k\right){>}_{k}-<S\left(i,j,k\right){>}_{k}$$

Where ^*^ denotes multiplication. Notably *I*_*Bliss*_*(i,j)* is defined in terms of the averages obtained for the wells *(i,0)*, *(0,j)*, and *(i,j)*. This index stems from the conventional Bliss independence assumption S(c_i_,c_j_ )= S(c_i_,0)S(0, c_j_) where S denote S(c_i_, c_j_) survival for drug combination (i,j) at concentrations (c_i_, c_j_).

The bootstrap intervals are obtained by simulating *B=1000* new batches of *K* experiments as follows. For each batch *b=1,2,…, B* a set of *K* different plates are simulated by generating survival indices according to the following procedure:

1. For well *(i,j)* on plate *k*, draw with replacement a random error from the pool of residuals *e*_*s*_*(i,j,k)* and change its sign with probability *50%* (assuming symmetric distribution) to produce the simulated error *e*_*b*_*(i,j,k)*.

2. Generate the bootstrap survival index *S*_*b*_*(i,j,k)* as *S*_*b*_*(i,j,k) = <S(i,j,k)>*_*k*_*+e*_*b*_*(i,j,k)*

3. Repeat 1 and 2 for all wells and plates (indices *i, j* and *k*).

4. Repeat 1-3 *B* times.

Using the resulting simulated values *S*_*b*_*(i,j,k)*, the corresponding bootstrap based synergy indices are determined as the difference (*b=1,2,…B*):

(4) $$I_{Bliss}\left(b,i,j\right)=<S_{b}\left(0,j,k\right){>}_{k}*<S_{b}\left(i,0,k\right){>}_{k}-<S_{b}\left(i,j,k\right){>}_{k}$$

This results in a set of B different bootstrap synergy indices {*I*_*Bliss*_*(b,i,j)*} for each well *(i,j)* that finally is described more compactly by a bootstrap interval (BI) *[a*_*ij*_*,b*_*ij*_*]* where the lower bound *a*_*ij*_ is defined as the *2.5%* percentile of the distribution in the set {*I*_*Bliss*_*(b,i,j)*} and *b*_*ij*_ denotes the corresponding *97.5%* percentile.

#### Loewe bootstrap interval (BI) calculations

BIs for deviations from Loewe additivity are calculated in the same way as above for deviations from Bliss independence. The only difference is the synergy index *I*_*Loewe*_*(i,j)* for well *(i,j)* which is defined differently here. Let *R*_*A*_*(c*_*A*_ *)* and *R*_*B*_*(c*_*B*_ *)* denote the individual concentration-response curves for the two drugs A and B, respectively. Under the Loewe additivity model, if the concentration combination *(c*_*A*_*, c*_*B*_ *)* yields the survival level s, then the following equation is satisfied, which corresponds to a straight line (linear isobole):

(5) $$\frac{c_{A}}{R_{A}^{-1}\left(S\right)}+\frac{c_{B}}{R_{B}^{-1}\left(S\right)}=1$$

Here $R_{A}^{-1}\left(s\right)$ and $R_{B}^{-1}\left(s\right)$ denote the corresponding inverse functions evaluated at the survival index value s. Notably each of these two values corresponds to a single drug concentration yielding the survival level s. The equation is often written in as $\frac{c_{A}}{D_{A}}+\frac{c_{B}}{D_{B}}=1$ where $D_{A}=R_{A}^{-1}\left(s\right)$ and $D_{B}=R_{B}^{-1}\left(s\right)$ showing perhaps more clearly that the survival is constant along the linear isoboles (straight lines) defined by this equation. The expression in equation (5) can also be used to formulate a response surface model (RSM) expressing the expected response value *R(c*_*A*_*, c*_*B*_ *)* for the concentration combination *(c*_*A*_*, c*_*B*_ *)* as [18]:

(6) $$R\left(c_{A,}c_{B}\right)=\begin{array}{c} \mathrm{argmin}\\ s \end{array}\left|\frac{c_{A}}{R_{A}^{-1}\left(S\right)}+\frac{c_{B}}{R_{B}^{-1}\left(S\right)}-1\right|$$

Here *argmin* denotes the mathematical operator which finds and returns the minimum value of its input across all plausible values of the parameter of interest, in this case the survival level s. Notably, this response surface equation is of course only valid when $R_{A}^{-1}\left(s\right)$ and $R_{B}^{-1}\left(s\right)$ are well defined for all values of *s*. Notably, this is not the case when at least one of the drugs has a partial response (survival level) saturating at some plateau where *s>0*. Thus for such cases the Loewe model is not defined for all concentration pairs *(c*_*A*_*, c*_*B*_ *)*.

The use of (5) to represent surfaces as well as more explicit parametric RSM to study Loewe predictive effects is well established [19, 21]. Also other approaches to RSM for synergy analysis have been proposed based on smoothing, interpolation, and non-parametric statistics [22-25].

Let *c*_*A*_*(i)* and *c*_*B*_*(j)* denote concentrations of drug A and B in well *(i,j)*, respectively. Then the predicted survival in well *(i,j)* according to (6) is $S_{Loewe}\left(i,j\right)=R\left(c_{A}\left(i\right),c_{B}\left(j\right)\right)\cdot$ This predicted value is compared with the average of all the actual experimental values observed yielding the Loewe synergy index *I*_*Loewe*_*(i,j)* defined as:

(7) $$I_{Loewe}\left(b,i,j\right)=S_{Loewe}\left(b,i,j\right)-<S_{b}\left(i,j,k\right){>}_{k}$$

Notably, the calculations in (6) require estimation of the inverse of the single drug concentration-response curves. This was achieved as we did in our previous work^18^ by approximating the concentration-response curves by the simple classical Hill model R(c) = 1/(1+(c/c_50_)^H^) where the two parameters H and c_50_ are estimated jointly using least squares fitting. In this case, the start values for the two parameters of the Hill models were obtained by fitting the Hill model using only two experimental data points making it possible to derive explicit formulas that directly yield the desired start values. Then least squares fitting was performed using a conventional steepest decent algorithm with a built-in adaptive step length procedure to achieve a refined fit of the Hill model to the whole set experimental data points. To obtain a self-contained solution and have full control of all implementation related details, the least squares fitting was implemented as part of COMBIA, instead of using some external already available R implementation.

Using the resulting simulated values *S*_*b*_*(i,j,k)* generated as described in the previous subsection, the corresponding bootstrap based synergy indices are determined as (*b=1,2,…B*):

$$S=\frac{f_{\mathrm{exposed}}-f_{\mathrm{blank}}}{f_{\mathrm{control}}-f_{\mathrm{blank}}}$$

Here *s*_*Loewe*_*(b,i,j)* is the predicted Loewe response based on the single response data sets {*S*_*b*_*(0,j,k)*} and {*S*_*b*_*(i,0,k)*}generated in batch *b*. As in the previous subsection this results in a set of B different bootstrap synergy indices {*I*_*Loewe*_*(b,i,j)*} for each well *(i,j)* that finally is represented more compactly by a bootstrap interval (BI).

### BI for the well of maximum synergy – foundation for a global (omnibus) test

In practice one is often focused on the well showing the largest synergy in terms of having the largest value of the synergy index defined in equations (3) and (7), here denoted *I*_*max*_. To obtain bootstrap estimates for this particular synergy index the simulation results above can be used again with a slight modification of the subsequent analysis as follows.

For each batch *b=1,2,…,*B, the corresponding largest synergy value is determined, here denoted *I*_*max*_*(b).* Thus *I*_*max*_*(b)* is the largest value in the set *{I*_*Bliss*_*(b,i,j)}* for Bliss or in the set *{I*_*Loewe*_*(b,i,j)}* for Loewe. Notably, the actual well *(i*_*b*_*, j*_*b*_*)* which yields the maximum value in a particular batch b will typically not be the same for all batches. This results in a set of B different bootstrap synergy indices *{I*_*max*_*(b)}* that finally is represented by a BI *[a*_*max*_*,b*_*max*_*]* where the lower bound *a*_*max*_ is defined as the 2.5% percentile of the distribution in the set {I_max_(b)} and *b*_*max*_ denotes the corresponding 97.5% percentile. Using an equivalent procedure we also obtained a BI for *I*_*min*_ defined as the smallest value of the synergy index defined in equations (3) and (7).

By focusing on the maximum and minimum values, rather than on the individual wells, the associated BI is suitable for a global (omnibus) test. Thus by determining if these null (Bliss or Loewe) hypothesis based BIs include the actual *I*_*min*_ and *I*_*max*_ observed, or not, a global statistical test is performed. Compared to individual statistical testing for each well separately, a single global statistical test is attractive as it avoids the need for a typically too conservative (Bonferroni) correction for multiple testing.

### Definition and statistical detection of synergy

In this context synergy (antagonism) is said to occur when the synergy index defined in eq. (3) or eq.(8) is larger (smaller) than zero and the number of plates (identical experiments) is very large. However, since only *K=4* plates are used for each drug combination tested, there will be substantial experimental variability. Using the resampling approach described above BIs are obtained for the synergy index *I(i,j)* in each well *(i,j)* as well as for the maximum value *I*_*max*_ according to each batch of *K=4* plates/experiments. Synergy is said to be detected in a well *(i,j)* if the BI for the synergy index *I(i,j)* does not include the value zero. Notably these BIs should not be rigorously interpreted as conventional confidence intervals as they do not have the proper coverage properties [26, 27]. However they do represent and quantify the statistical uncertainty in a theoretically sound manner that only relies on weak/general assumptions about the underlying statistical distributions involved.

For the global (omnibus) test, the resampling distribution of maximum synergy values *I*_*max*_ assuming the Bliss or Loewe model to be true were calculated. For a test at significance level 2.5%, global synergy was said to be detected if the observed *I*_*max*_ was larger than the 97.5-percentile of the distribution. Similarly global (omnibus) antagonism was said to be detected when the observed *I*_*min*_ was smaller than the 2.5-percentile of minimum antagonistic values *I*_*min*_ under the null (Bliss or Loewe) hypothesis. A combination is said to be synergistic or antagonistic if it deviates from both Bliss and Loewe models as shown in the Table 1 and in Figures 1-6 with bold lines.

## SUPPLEMENTARY MATERIALS FIGURES AND TABLES



## References

[1] Cao Y, DePinho AR, Ernst M, Vousden K (2011). Cancer research: past, present and future. Nature Rev Cancer.

[2] Adams DJ (2012). The valley of death in anticancer drug development: a reassessment. Trends Pharmacol Sci.

[3] De PM, Hanahan D (2012). The biology of personalized cancer medicine: facing individual complexities underlying hallmark capabilities. Mol Oncol.

[4] Carrato A, Swieboda-Sadlej A, Staszewska-Skurczynska M, Lim R, Roman L, Shparyk Y, Bondarenko I, Jonker DJ, Sun Y, De la Cruz JA, Williams JA, Korytowsky B, Christensen JG (2013). Fluorouracil, leucovorin and irinotecan plus sunitinib or placebo in metastatic colorectal cancer: a randomized phase III trial. J Clin Oncol.

[5] Yap TA, Omlin A, de-Bono JS (2013). Development of therapeutic combinations targeting major cancer signaling pathways. J Clin Oncol.

[6] Schmoll HJ, Van CE, Stein A, Valentini V, Glimelius B, Haustermans K, Nordlinger B, van de Velde CJ, Balmana J, Regula J, Nagtegaal ID, Beets-Tan RG, Arnold D (2012). ESMO consensus guidelines for management of patients with colon and rectal cancer. A personalized approach to clinical decision making. Ann Oncol.

[7] Grothey A, Van CE, Sobrero A, Siena S, Falcone A, Ychou M, Humblet Y, Bouche O, Mineur L, Barone C, Adenis A, Tabernero J, Yoshino T (2013). Regorafenib montherapy for previously treated metastatic colorectal cancer (CORRECT): an international, multicentre, randomised, placebo-controlled phase 3 trial. Lancet.

[8] Prichard MN, Shipman C (1990). A three-dimensional model to analyze drug-drug interactions. Antiviral Res.

[9] http://www.uab.edu/images/pediatrics/ID/Webpage_Mark_Prichard.pdf, accessed 2014-12-12

[10] Bliss CI (1939). The toxicity of poisons applied jointly. Ann Appl Biol.

[11] Loewe S, Muischnek H (1926). Über Kombinationswirkungen. I. Mitteilung: Hilfsmittel der Fragestellung. Arch Exp Path Pharmakol.

[12] Loewe S (1953). The problem of synergism and antagonism of combined drugs. Arzneimittelforschung.

[13] Chou TC, Talalay P (1984). Quantitative analysis of dose-effect relationship: the combined effects of multiple drugs or enzyme inhibitors. Adv Enzyme Regul.

[14] Boik CJ, Narasimhan B (2010). An R package for assessing drug synergyism/antagonism. J Stat Software.

[15] Saul A, Fay PM (2007). Human immunity and the design of multi-component, single target vaccines. PLoS One.

[16] Mayer DL, Harasym OT, Tardi GP, Harasym LN, Shew RC, Johnstone AS, Ramsay CE, Bally BM, Janoff SA (2006). Ratiometric dosing of anticancer drug combinations: Controlling drug ratios after systemic administration regulates therapeutic activity in tumor-bearing mice. Mol Cancer Ther.

[17] Lancet JE, Cortes JE, Hogge DE, Tallman MS, Kovacsovics TJ, Damon LE, Komrokji R, Solomon SR, Kolitz JE, Cooper M, Yeager AM, Louie AC, Feldman EJ (2014). Phase 2 trial of CPX-351, a fixed 5:1 molar ratio of cytarabine/daunorubicin, vs cytarabine/daunorubicin in older adults with untreated AML. Blood.

[18] Kashif M, Andersson C, Åberg M, Nygren P, Sjöblom T, Hammerling U, Larsson R, Gustafsson MG (2014). A pragmatic definition of therapeutic synergy suitable for clinically relevant *in vitro* multicompound analyses. Mol Cancer Ther.

[19] Berenbaum MC (1989). What is synergy?. Pharmacol Rev.

[20] Lindhagen E, Nygren P, Larsson R (2008). The fluorometric microculture cytotoxicity assay. Nat Protoc.

[21] Greco WR, Bravo G, Parsons JC (1995). The search of synergy: A critical review from a response surface perspective. Pharmacological Rev.

[22] Carter WH, Gennings C, Staniswalis JG, Cambell ED, White KL (1988). A statistical approach to the construction and analysis of isobolograms. J Am College Toxicology.

[23] Novick SJ (2013). A simple test for synergy for a small number of combinations. Statistics in Medicine.

[24] Kelly C, Rice JR (1990). Monotone smoothing with application to dose-response curves and the assessment of synergism. Biometrics.

[25] Kong M, Lee JJ (2008). A semiparametric response surface model for assessing drug interaction. Biometrics.

[26] Efron B (1987). Better Bootstrap Confidence Intervals. J Am Stat Assoc.

[27] Chernickc RM, Labudde AR (2009). Revisiting Qualms about Bootstrap Confidence Intervals. Am J Math Managet Sci.

[28] Zhang X, Fryknäs M, Hernlund E, Fayad W, De Milito A, Olofsson MH, Gogvadze V, Dang L, Påhlman S, Schughart LA, Rickardson L, D'Arcy P, Gullbo J (2014). Induction of mitochondrial dysfunction as a strategy for targeting tumour cells in metabolically compromised microenvironments. Nat Commun.

[29] Di Nicolantonio F, Martini M, Molinari F, Sartore-Bianchi A, Arena S, Saletti P, De Dosso S, Mazzucchelli L, Frattini M, Siena S, Bardelli A (2008). Wild-type BRAF is required for response to panitumumab or cetuximab in metastatic colorectal cancer. J Clin Oncol.

[30] He L, Kulesskiy E, Saarela L, Turunen L, Wennerberg K, Aittokallio T, Tang J (2016). Methods for high-throughput drug combination screening and synergy scoring. Springer Protocol.

