# Supplementary material for: Bliss and Loewe interaction analyses of clinically relevant drug combinations in human colon cancer cell lines reveal complex patterns of synergy and antagonism
Source: Oncotarget. 2017 Oct 19;8(61):103952–67. doi: 10.18632/oncotarget.21895 (PMC5732778; doi:10.18632/oncotarget.21895)
Supplement: Supplementary file 1 [file oncotarget-08-103952-s001.pdf]

Bliss and Loewe interaction analyses of clinically relevant drug combinations in human colon cancer cell lines reveal complex patterns of synergy and antagonism

SUPPLEMENTARY MATERIALS

Supplementary material (Appendix)

This supplement contains details regarding the combinations showing joint Bliss and Loewe synergy or antagonism in the different model systems (Supplementary Figures 1-6). The first Supplementary Figure 1 is discussed in some detail in order to explain how the remaining Supplementary Figures 2-6 should be analyzed in the same manner although the same kind of detailed discussion is not provided for them. Supplementary Table 1 shows the summary of Bliss and Loewe individual synergy analyses for the 15 combinations tested in 6 cell lines and Table 2 shows the summary results of Bliss and Loewe antagonism analyses.

S1: Details regarding combinations showing joint Bliss and Loewe synergy or antagonism in HCT116

Four combinations, shown in the first column of Supplementary Figure 1 are found to be synergistic (panels A-D) or antagonistic (panels E-H) according to the joint Bliss and Loewe analysis. They are represented by the solid lines in Figure 1 in the main text. Each combination has two detailed figures, one on the left (2nd column) corresponding to Bliss analysis and one on the right (3rd column) corresponding to Loewe analysis of same experimental data. More specifically, panels A, C, E and G present the Bliss analyses and panels B, D, F and H present the de-convoluted graphs of Loewe analyses for combinations erlotinib + irinotecan, VLX600 + oxaliplatin, erlotinib + sunitinib and sunitinib + 5FU, respectively. For example in panel A, Bliss analysis results for the HCT116 cell line after exposure to erlotinib and irinotecan are shown. Each vertex in panel A corresponds to a concentration combination; yellow-red spots (peaks) represent synergy (non-overlapping 95% BIs) while cyan-blue spots (depressions) represent antagonism (non-overlapping 95% BIs). All concentration pairs with 95% BIs covering zero are colored green (zero). The color bar on the right reflects the synergy index defined in (3). Thus, in panel A, the combination erlotinib and irinotecan produces a prominent synergistic spot at 64-128µM of irinotecan and 25-100µM of erlotinib, while spot of synergy values in panel B (Loewe analysis of same data) was found at 16-128µM of irinotecan and 12.5-100µM of erlotinib.

The I_{max} (see section of COMBIA calculations for details) for panel A is 28.7 and the corresponding BI is (7.1, 14.3). For panel B, I_{max} is 41.2 with the corresponding BI (4.6, 9.3). Since in both cases, the observed I_{max} values are much greater than the upper bounds on the respective BIs, synergy is said to be detected according to both Bliss and Loewe global synergy analyses. This is represented by a solid line in Figure 1 of the main text. Similarly, the combination VLX600 + oxaliplatin was also synergistic while the other two combinations erlotinib + sunitinib and sunitinib + 5FU show antagonism according to the same global analysis.

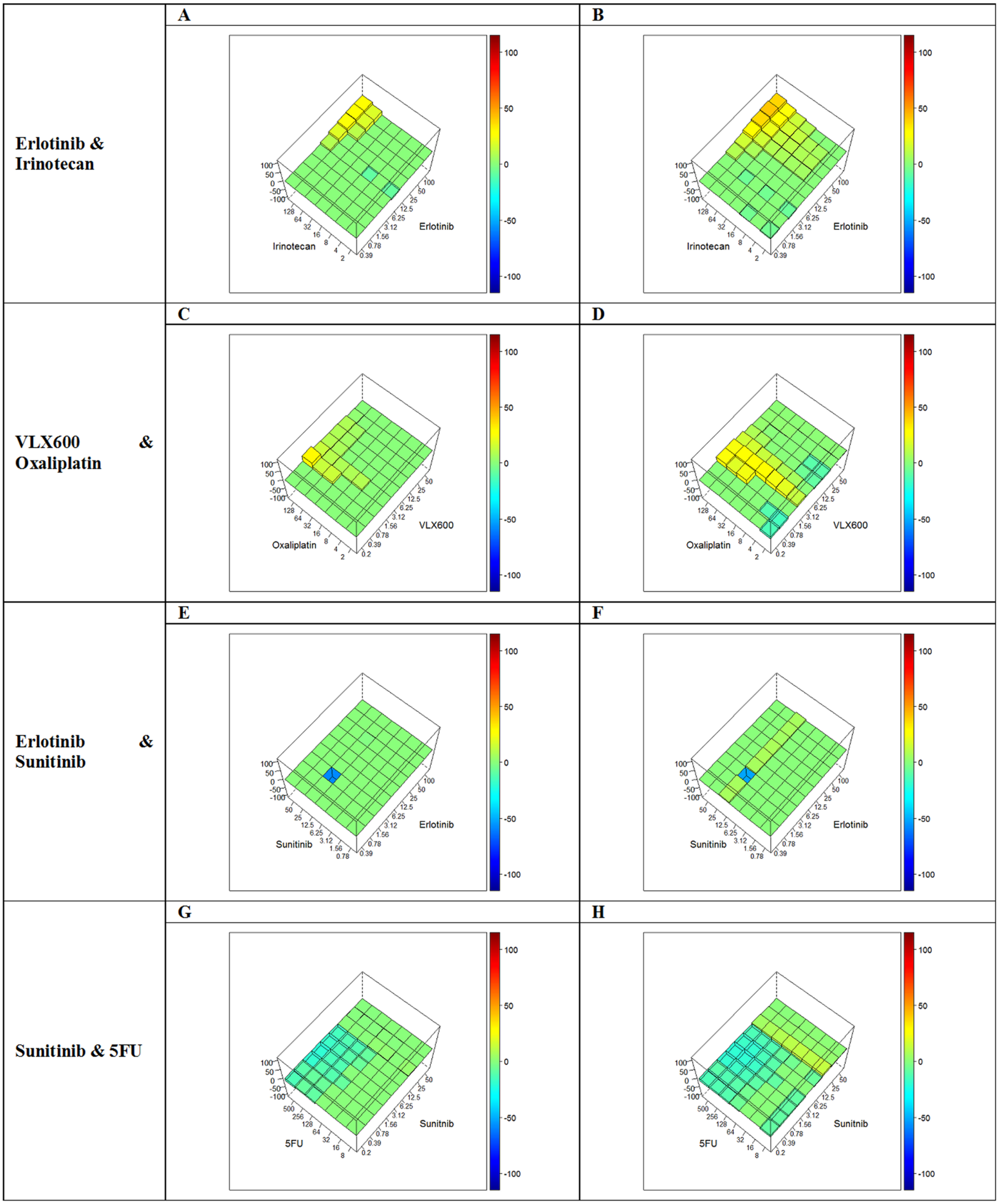

Supplementary Figure 1: De-convoluted combinations showing joint Bliss and Loewe synergy or antagonism in HCT116. All these four combinations are represented by the solid lines in Figure 1 of the main manuscript. Combinations erlotinib + irinotecan (panels **A** and **B**) and VLX600 + oxaliplatin (panels **C** and **D**) are synergistic while combinations erlotinib + sunitinib (panels **E** and **F**) and sunitinib + 5FU (**G** and **H**) are antagonistic.

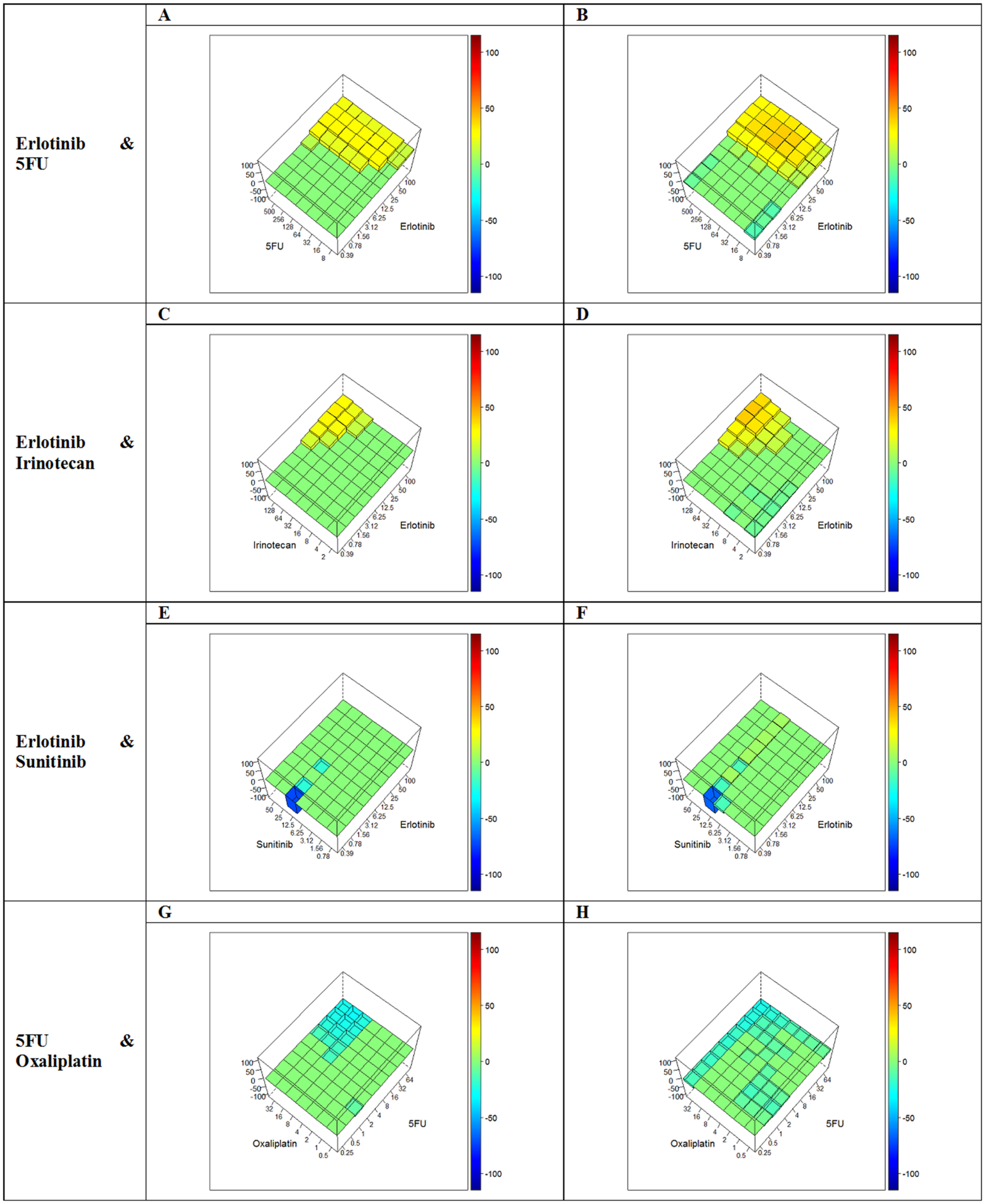

Supplementary Figure 2: Details regarding combinations in cell line HCT116KRAS/- showing joint Bliss and Loewe synergy or antagonism. All these four combinations are represented by the solid lines in Figure 2 of the main manuscript. The combinations erlotinib + 5FU (panels **A** and **B**) and erlotinib + irinotecan (panels **C** and **D**) are synergistic while the combinations erlotinib + sunitinib (panels **E** and **F**) and 5FU + oxaliplatin (**G** and **H**) are antagonistic.

Erlotinib & Sunitinib

A

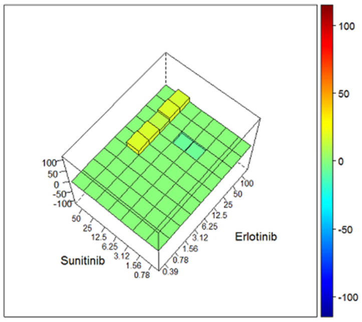

B

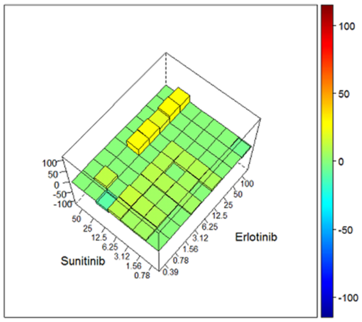

VLX600 & Irinotecan

C

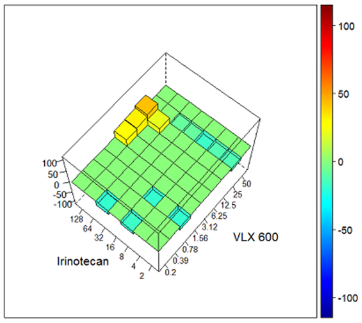

D

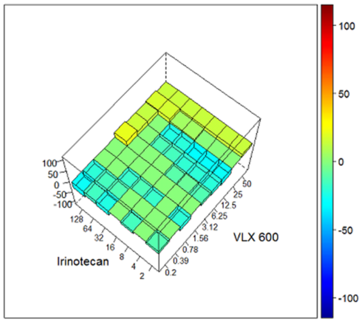

Erlotinib & Oxaliplatin

E

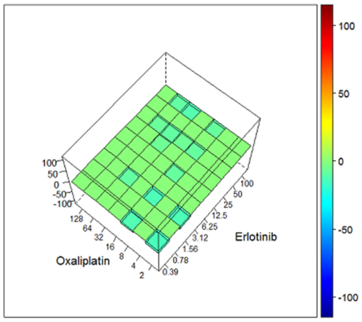

F

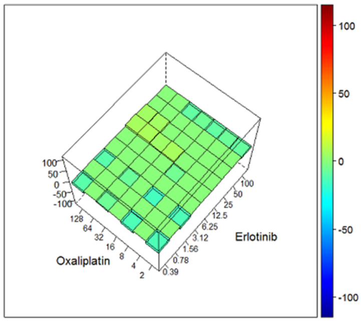

5FU & Oxaliplatin

G

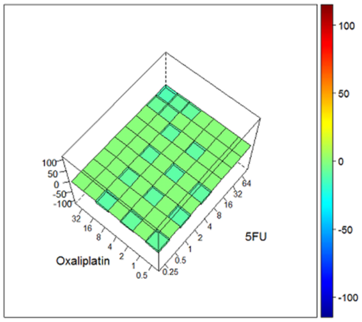

H

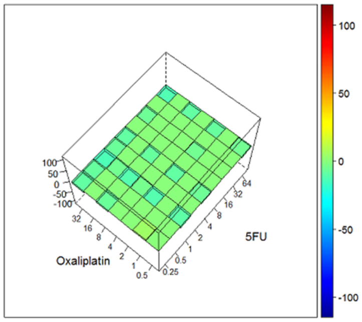

Supplementary Figure 3: Details regarding the combinations showing joint Bliss and Loewe synergy or antagonism in DLD-1. In Figure 3 of the main text all these combinations are represented by the solid lines. The combinations erlotinib + sunitinib (panels **A** and **B**) and VLX600 + irinotecan (panels **C** and **D**) are synergistic while the combinations erlotinib + oxaliplatin (panels **E** and **F**) and 5FU + oxaliplatin (**G** and **H**) are antagonistic.

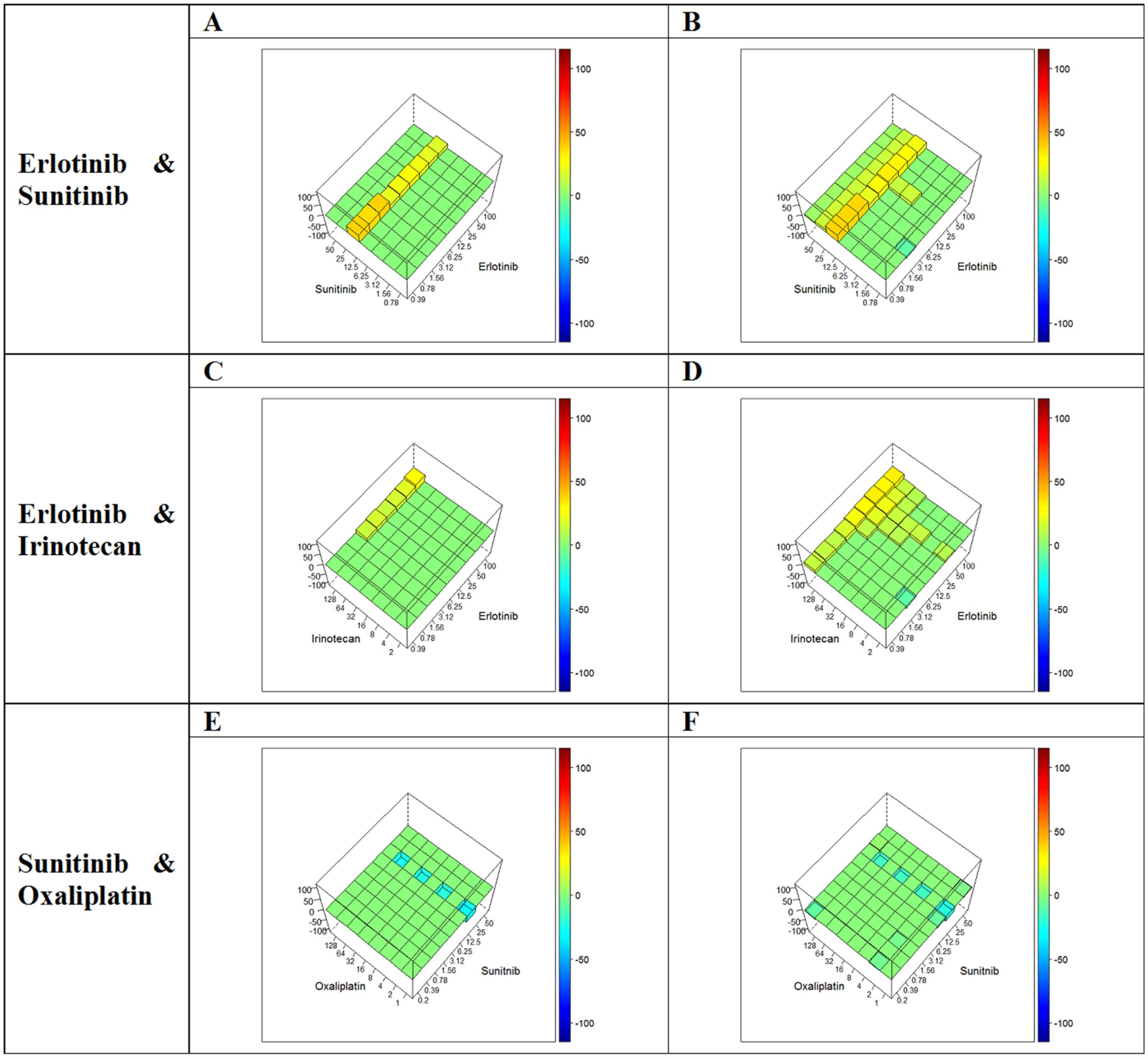

Supplementary Figure 4: Details regarding the combinations in cell line DLD-1KRAS/- showing joint Bliss and Loewe synergy or antagonism. The combinations erlotinib + sunitinib, erlotinib + irinotecan and sunitinib + oxaliplatin are represented by the solid lines in Figure 4 in the main text. Panels **(A-D)** show synergistic combinations and panels **(E)** and **(F)** are showing an antagonistic combination.

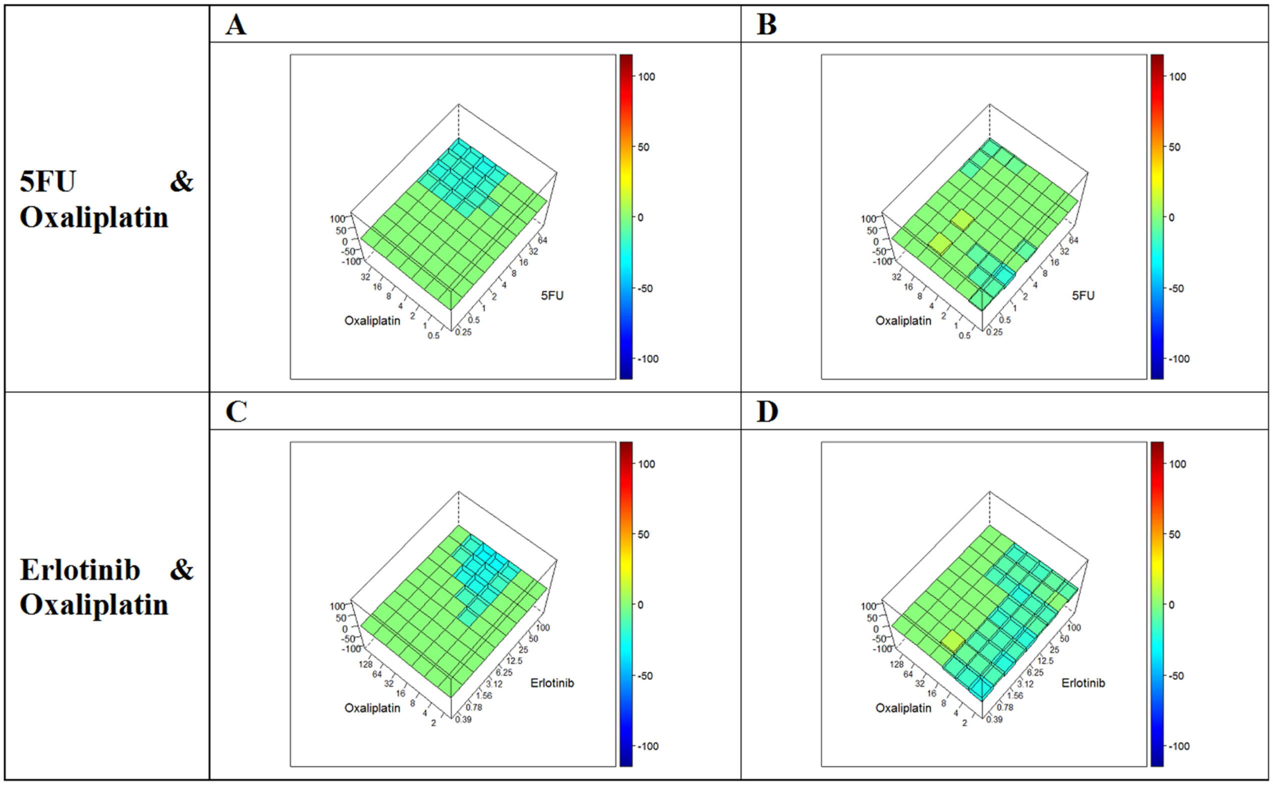

Supplementary Figure 5: Details regarding combinations showing joint Bliss and Loewe synergy or antagonism in RKO. The two combinations 5FU + oxaliplatin and erlotinib + oxaliplatin are represented by the solid lines in Figure 5 of the main text. Their detailed graphs (panels **A-D**) show they are antagonistic and no synergistic combination was found for the RKO.

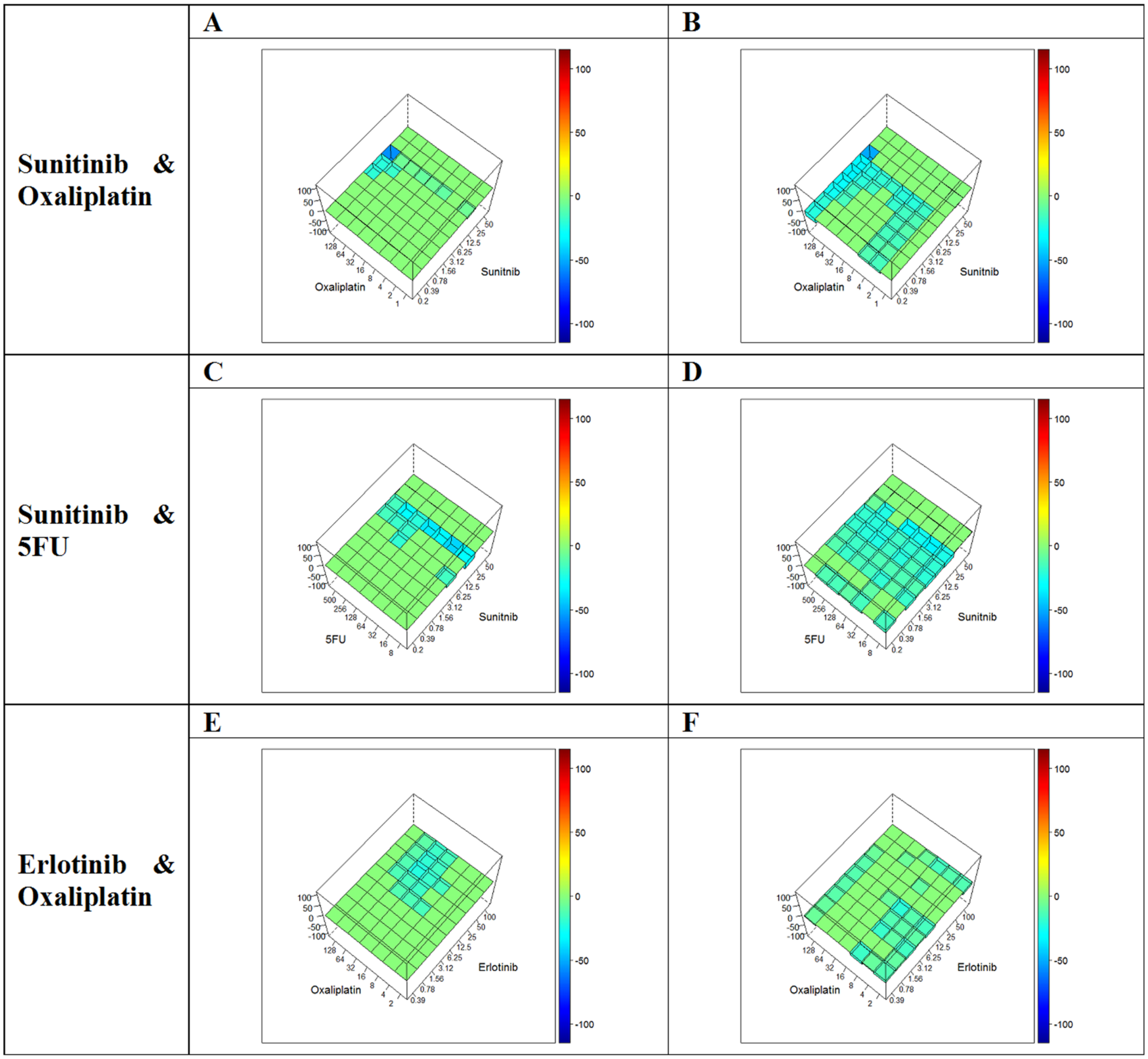

Supplementary Figure 6: Details regarding combinations showing joint Bliss and Loewe synergy or antagonism in RKOBRAF/-/-. Three combinations (sunitinib + oxaliplatin, sunitinib + 5FU and erlotinib + oxaliplatin) are shown as solid lines in Figure 6 of the main text. The corresponding detailed graphs presented here (panels **A-F**) show they are antagonistic (blue color). No synergistic combination was found for the RKOBRAF/-/-.

Supplementary Table 1: Summary of Bliss and Loewe synergy analyses of drug combinations tested in 6 cell lines

Combination	HCT116		HCT116KRAS/-		DLD-1		DLD-1KRAS/-		RKO		RKOBRAF/-/-	
	Bliss Syn.	Loewe Syn.	Bliss Syn.	Loewe Syn.	Bliss Syn.	Loewe Syn.	Bliss Syn.	Loewe Syn.	Bliss Syn.	Loewe Syn.	Bliss Syn.	Loewe Syn.
Erlotinib & Sorafenib												
Erlotinib & Sunitinib					X	X	X	X		X		X
5FU & Irinotecan		X		X				X				
5FU & Oxaliplatin												X
Sorafenib & 5FU												
Sorafenib & Irinotecan				X								X
Sorafenib & Oxaliplatin												
Sunitinib & Irinotecan		X										
Sunitinib & Oxaliplatin				X								
Sunitinib & 5FU		X				X						
Erlotinib & 5FU		X	X	X				X				X
Erlotinib & Irinotecan	X	X	X	X			X	X				X
Erlotinib & Oxaliplatin		X				X						
VLX600 & Irinotecan		X		X	X	X		X				X
VLX600 & Oxaliplatin	X	X		X		X		X		X		X

Each element in the table, which corresponds to one particular drug combination, cell line and synergy calculation model, is marked by "X" when there exists a synergy (occurs when the bootstrap interval of I_{max} under the Bliss or Loewe null model does not include observed I_{max}).

Supplementary Table 2: Summary of Bliss and Loewe antagonism analyses of drug combinations tested in 6 cell lines

Combination	HCT116		HCT116KRAS/-		DLD-1		DLD-1KRAS/-		RKO		RKOBRAF/-/-	
	Bliss Ant.	Loewe Ant.	Bliss Ant.	Loewe Ant.	Bliss Ant.	Loewe Ant.	Bliss Ant.	Loewe Ant.	Bliss Ant.	Loewe Ant.	Bliss Ant.	Loewe Ant.
Erlotinib & Sorafenib						O						
Erlotinib & Sunitinib	O	O	O	O								
5FU & Irinotecan						O		O				
5FU & Oxaliplatin			O	O	O	O		O	O	O		
Sorafenib & 5FU						O		O		O		O
Sorafenib & Irinotecan		O		O		O						O
Sorafenib & Oxaliplatin						O		O		O		O
Sunitinib & Irinotecan						O						O
Sunitinib & Oxaliplatin		O		O		O	O	O		O	O	O
Sunitinib & 5FU	O	O		O		O		O			O	O
Erlotinib & 5FU				O		O		O				
Erlotinib & Irinotecan												O
Erlotinib & Oxaliplatin		O			O	O			O	O	O	O
VLX600 & Irinotecan				O		O		O				O
VLX600 & Oxaliplatin		O				O		O		O		O

Each element in the table, which corresponds to one particular drug combination, cell line and synergy calculation model, is marked by “O” when there exists antagonism (occurs when bootstrap interval of I_{min} does not include observed I_{min}).
